# MetaTIS: a tool to predict cognate and near-cognate translation initiation sites in human

**DOI:** 10.1093/nargab/lqag100

**Published:** 2026-08-24

**Authors:** Aram Papazian, Volkhard Helms

**Affiliations:** Center for Bioinformatics, Saarland Informatics Campus, Saarland University, Saarbruecken 66041, Germany; Center for Bioinformatics, Saarland Informatics Campus, Saarland University, Saarbruecken 66041, Germany

## Abstract

Ribosomes typically commence translation at a methionine-encoding AUG codon flanked by a so-called Kozak region, a short nucleic acid motif that serves as an initiation site in humans. Though, the characteristic AUG start codon of an mRNA is not always effective in initiating translation. Near-cognate codons differing from AUG by one nucleotide may also be recognized as start sites. Several types of ribosomal profiling techniques have been developed that elucidate active translation initiation sites (TIS) that enable training of computational models to predict both cognate and near-cognate TIS using mRNA sequence features. Here, a meta-model termed MetaTIS was implemented by combining outputs of genomic and protein language models fine-tuned on Ensembl annotations of transcripts and five different TIS datasets. The model proficiently differentiates between spurious and true TIS in four distinct test sets, for both AUG and non-AUG instances. Most important for translation initiation based on one of the base model outputs was the Kozak sequence context and a region further upstream in the 5′UTR [−12, −10]. MetaTIS is available as a webserver at https://service2.bioinformatik.uni-saarland.de/metatis/, a tool that accurately predicts TIS for AUG and nine near-cognate start codons.

## Graphical Abstract

**Graphical Abstract:**
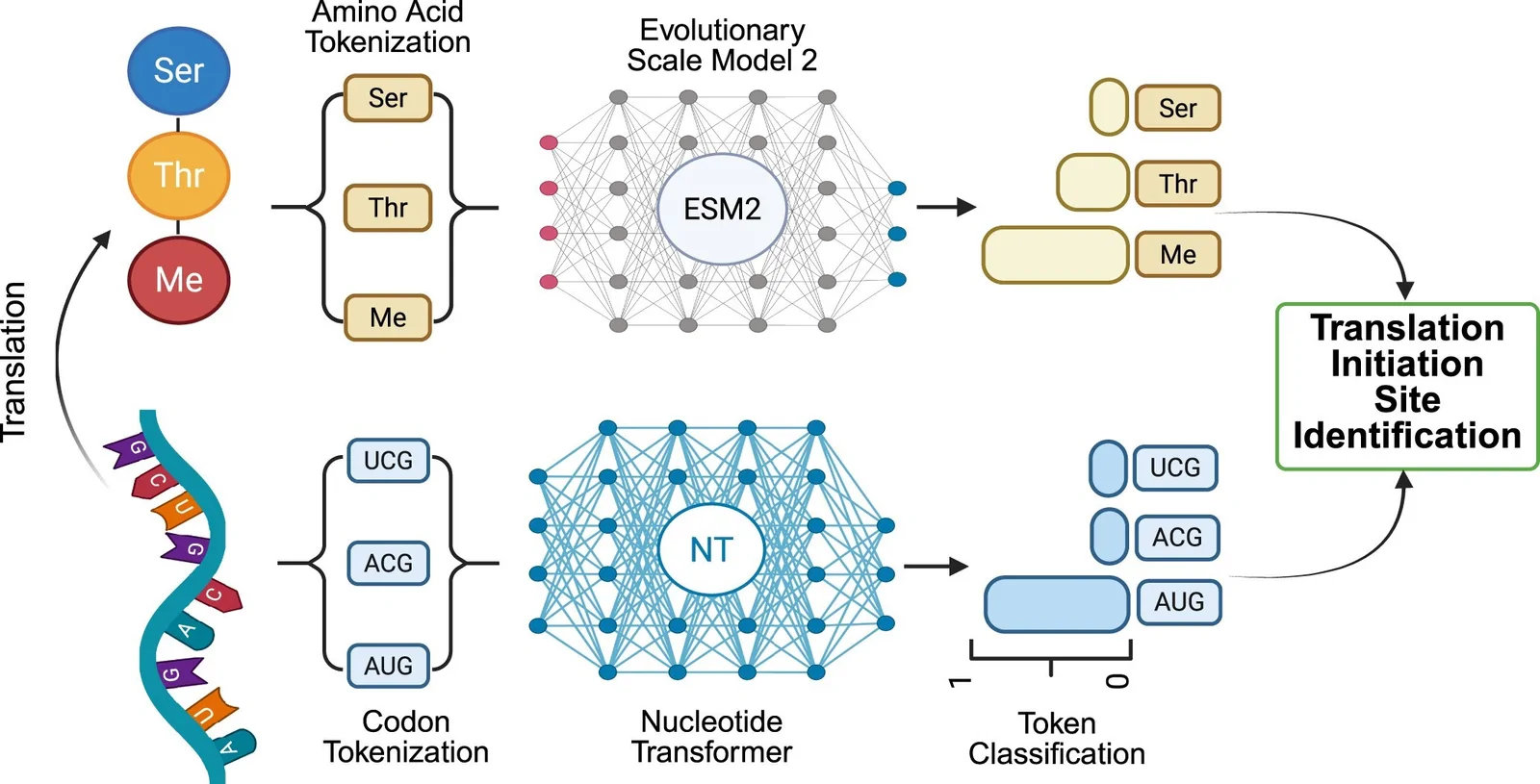

## Introduction

Ribosomal translation of messenger RNAs is one of many core cellular processes observed across all kingdoms of life. In eukaryotes, translation initiation is dependent on the assembly of complex eukaryotic initiation factor (eIF) machinery and proceeds in several stages via the 40S, 43S, 48S, and 80S initiation complexes [1]. Throughout the mid-to-late 1980’s, Kozak contributed unprecedented insight into the fundamental characteristics of start codons, of whom the Kozak region shares its namesake [2]. As exemplified in the translation of the preproinsulin gene, Kozak revealed that the third upstream nucleotide and first downstream nucleotide, with respect to the AUG-start codon, are critical for translation initiation. Additionally, further sequence features implied in start codon recognition have been determined in high resolution. As shown in translation efficiency studies implementing GFP variants, there exist several possible nucleotide variations in the −6 to +5 positions around AUG codons [3] and non-cognate start codons [4]. Aside from the canonical AUG codon, alternative near-cognate codons, occurring mostly as CUG and GUG, may also be used as start codons; these variants are often conserved between *Homo sapiens* and *Mus musculus* [5].

Large-scale translatomic screening via ribosomal profiling (Ribo-seq) is a powerful *in vivo* assay commonly employed to investigate which mRNA sequences are actively translated into proteins, giving the investigator a “freeze frame” of the translatome landscape as ribosomal activity is brought to a halt [6]. However, Ribo-seq alone is not capable of directly identifying translation initiation sites (TIS). Consequently, newer approaches, such as global translation initiation sequencing [7] and quantitative translation initiation sequencing (QTI-seq), [8] have been developed with the aim of determining start codon positions *in vivo* at a single nucleotide resolution. Recent studies provide more holistic insight into TIS detection, incorporating mass spectrometry, Ribo-seq, selective translation complex profile sequencing (Sel-TCP-seq), and several Clustered Regularly Interspaced Short Palindromic Repeats (CRISPR) based screenings to identify novel short peptides in the human genome [9, 10]. In 2022, Mudge *et al.* outlined a community-led effort incorporating data from Ensembl/GENCODE [11], the HUGO Gene Nomenclature Committee (HGNC) [12], UniProtKB [13], the Human Proteome Organization/Human Proteome Project [14], and PeptideAtlas [15] to develop a standardized catalogue of human Ribo-seq open reading frames (ORFs) [16].

Shortly after these methods were developed, bioinformatics machine learning models trained on their outputs emerged, prompting the prediction of putative start codons based on their proximal sequence features. Of these models, the webserver PreTIS [17] predicts the translation efficiency of cognate and all near-cognate codons differing from AUG by one nucleotide; the model performs at an accuracy of 80% and uses a linear regression model trained on Ribo-seq data. A deep learning predictor composed of recurrent and convolution layers, TITER [18], which was trained on QTI-seq data [8], uses as input the 100 nucleotides upstream and downstream of the start codon. Similarly, Gleason *et al.* [19] presented a random forest classifier with a reported accuracy of 85%, based on the 10 nucleotides upstream and downstream of the start codon. Additionally, two transformer-based models that predict solely canonical AUG TIS were developed, as opposed to previous methods. The first of which, TISTransformer [20], processes the full sequence of the transcript to impute TIS presence at each nucleotide position; the model was trained on the entire Ensembl assembly of the human genome, achieving an area under the precision recall curve (PRAUC) of ~0.83. That study also highlighted the prowess of the transformer architecture in TIS detection by comparing it against previous approaches including TITER [18]. The second transformer-based model, NetStart 2.0 [21], utilized the protein language model “Evolutionary Scale Modeling v2” (ESM2) [22] to predict AUG TIS for a diverse range of eukaryotic species, including *Homo sapiens*. Most recently, TranslationAI [23] and TRANSAID [24] were developed to predict both the TIS and translation termination site of a transcript. TranslationAI is a 32-layer dilated convolutional neural network that predicts canonical and non-canonical TIS in human. TRANSAID uses a hierarchical deep learning architecture that combines a neural network for sequence analysis with a downstream biological feature scoring system and predicts canonical TIS.

ESM2 was designed based on the BERT Framework [25] and pretrained on the Uniprot dataset [13] using masked language model learning. Nucleotide transformers (NT) are state-of-the-art foundation models pretrained on DNA sequences from multiple species [26]; these models yield context-specific representations of nucleotide sequences, which allow for accurate predictions in several genomic applications. In this study, both the ESM2 and NT models were fine-tuned to predict both cognate and near-cognate TIS using ORFs curated from five relevant studies, as well as the Ensembl assembly of the human genome, GRCh38.p14. To combine the benefits of fine-tuned genomic and protein language models and to calibrate prediction probabilities, a meta-model termed MetaTIS was trained on the outputs of both models. Formidable performance was demonstrated when compared to previous approaches, implementing MMSeqs2 and DataSail to avoid data leakage [27, 28]. Our models were evaluated on external test sets and compared to the performance of alternative tools. Also, the integrated gradients of the flanking regions were analyzed to identify features promoting translation initiation. This approach is not only highly practical in novel protein discovery but may also help in detecting critical non-canonical TIS sites linked to multiple diseases.

## Materials and methods

### Data preparation

Annotated TIS were retrieved from the Ensembl assembly of the human genome, particularly GRCh38.p14 [11]. To resolve unannotated TIS, we referred to the findings of five benchmark studies: Gao *et al.* developed QTI-seq, which successfully identified TIS in HEK cells [8], Chen *et al.* discovered hundreds of uORFs derived by employing mass spectrometry, ribosome profiling, and several CRISPR-based screens [9], Mudge *et al.* created a standardized catalogue of human Ribo-seq ORFs [16], Rodriguez *et al.* found evidence of 192 translated upstream regions, most of which producing protein isoforms with extended N-terminal ends [29], and Ichihara *et al.* developed the TIS detection by translation complex analysis (TISCA) protocol [10]. Combining GTI-, Ribo-, and Sel-TCP-seq, TISCA was utilized to identify canonical and non-canonical TIS in HEK293 cells. The number of AUG and non-AUG TIS present in each dataset is mentioned in Table 1.

**Table 1.** The total number of AUG and non-AUG TIS retrieved from each source

Source	AUG TIS	Non-AUG TIS
Ensembl assembly [11]	83 522	34 086
Gao *et al.* [8]	6485	5876
Chen *et al.* [9]	2597	313
Mudge *et al.* [16]	3396	0
Rodriguez *et al.* [27]	11	160
Ichihara *et al.* [10]	29 781	12 263
Total without duplicates	100 192	50 331

By combining these datasets compiled by different methodologies, a comprehensive list of canonical and non-canonical TIS was obtained that is not reliant on one particular technique. We treated each detected TIS as equivalent without differentiating between sources. The rationale for this was to learn a general decision boundary applicable across all considered sources. Effectively, within this framework, a model can be trained to predict whether a transcript position can be classified as a TIS or non-TIS. Non-TIS include all positions in the transcript not found to be a TIS in any of the aforementioned datasets. Intriguingly, many transcripts were found to possess multiple TIS. These TIS datasets are made available on https://github.com/AramPapaz/MetaTIS.

### Fine tuning

**Figure 1.**
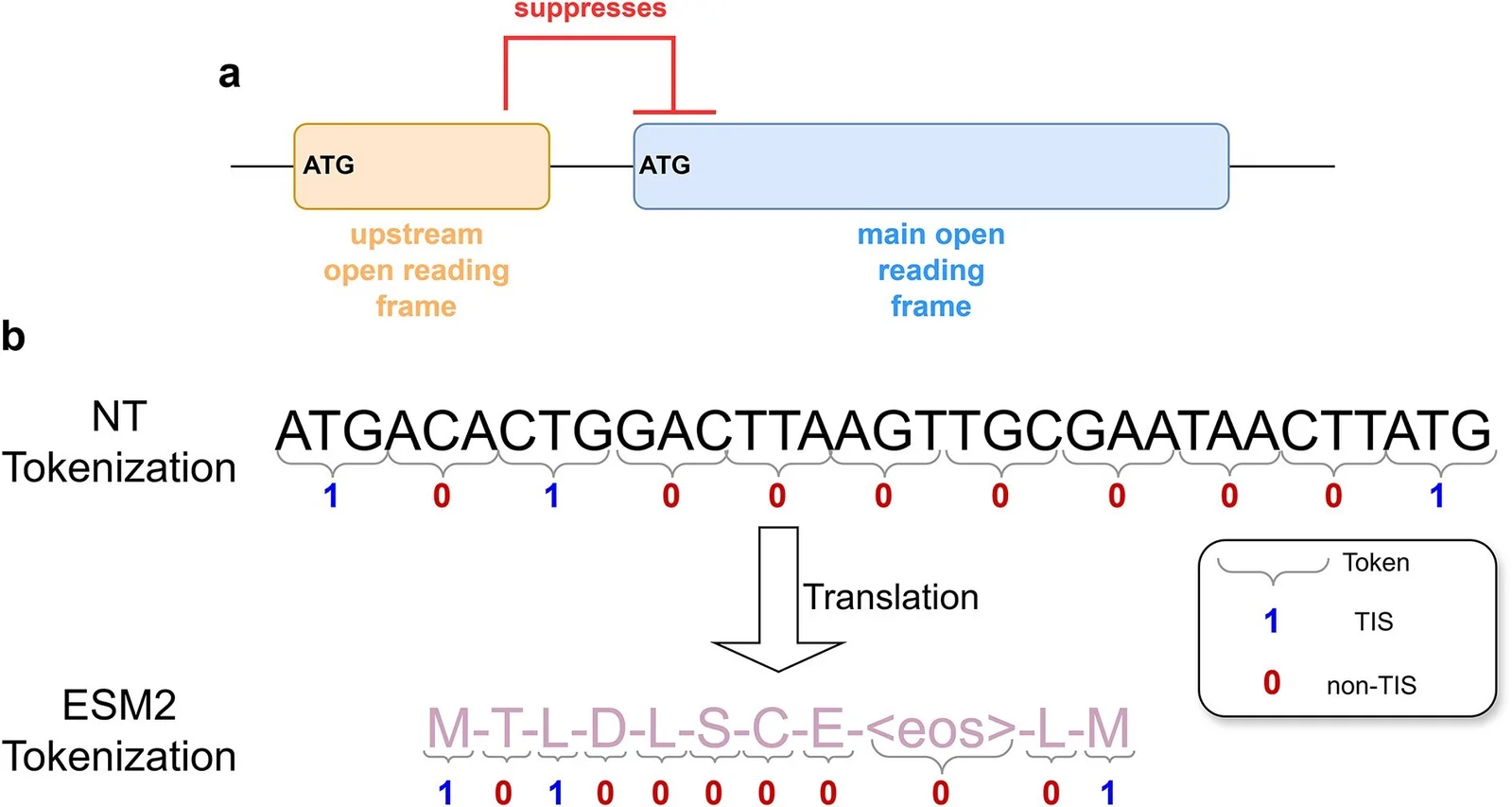
(**a**) The presence of an upstream ORF can suppress the translation of the main ORF. (**b**) Tokenization approaches for the NT and ESM2 models. The values listed below the sequences represent the labels of each token. The “<eos>” token symbolizes the end of a sentence; this token was used during translation of stop codons.

The models utilized in this study include an NT composed of 50 million parameters with 3-mer tokenization [30], as well as an ESM2 model composed of 35 million parameters and 12 layers. These models were fine-tuned for a token classification task using the Low-Rank Adaptation (LoRA) approach [31], such that a positive codon 3-mer token and a positive amino acid token symbolize a true TIS for the NT and ESM2 models, respectively. Token classification was selected as the task for the models to consider the presence of an upstream TIS when predicting a TIS further downstream. In fact, the presence of an upstream TIS has been demonstrated to significantly hinder the translation of downstream start codons [32]. Figure 1a visualizes this scenario. To implement this within an amino acid sequence, the stop codons UAG, UAA, and UGA were translated to an “<eos>” token, which represents the end of a sentence, as illustrated in Fig. 1b where thymine was used as a proxy for uracil. Ultimately, this tokenization approach was performed on all three reading frames.

LoRA was applied to the query, key, value, and dense modules with a rank of 8, a scaling factor of 16, and a dropout rate of 0.05. The models were trained using focal loss, which is illustrated in the equations below:


\begin{eqnarray*}
{p}_t = \left\{ {\begin{array}{@{}*{1}{c}@{}} {{p}_i\ \mathrm{ if}\ \mathrm{label} = 1}\\ {\left( {1 - {p}_i} \right)\ \mathrm{ if}\ \mathrm{label} = 0} \end{array}} \right.,
\end{eqnarray*}

\begin{eqnarray*}
\mathrm{Focal}\ \mathrm{loss} = {\left( {1 - {p}_t} \right)}^\gamma \log \left( {{p}_t} \right).
\end{eqnarray*}

Here, ${p}_i$ is the predicted probability of the ${i}{\mathrm{ th}}$ observation being in class 1, such that *γ * is the focusing parameter prioritizing more complex examples over easily discernible ones.

This loss was used to address class and codon imbalances. Note that, focal loss downweights well-classified instances and upweights hard-to-classify ones, such as near-cognate TIS, increasing their contribution to the training signal and improving the model’s ability in classifying them. The best model was selected such that it possessed the lowest loss on the validation set. For both models, a focusing parameter of 2, a learning rate of 2e^−4^, a cosine learning schedular, and warmup ratio of 0.1 were used. For ESM2, a weight decay of 0.01 was also used. Both models were trained for 20 epochs. The training runs were conducted using *Hugging Face Transformers version 4.55.3* [33], *PEFT version 0.17.1* [34], and *torch version 2.8.0 + cu129* [35].

### Meta model

To aggregate the prediction probabilities of the fine-tuned NT and ESM2 models, a meta-learner using logistic regression (LR) trained on the validation set was developed. Given the extreme class imbalance, balanced class weights were applied to the LR model to ensure high sensitivity toward the minority positive class. While this approach successfully maximized the precision-recall area under the curve (PRAUC), it introduces a systematic overconfidence in the output probabilities. To rectify this, a five-fold cross-validated isotonic regression calibration layer was implemented using *CalibratedClassifierCV* from *scikit-learn version 1.7.2* [36]. This non-parametric approach was chosen due to the large amount of the data, allowing for correct mapping of distorted scores back to true empirical frequencies. Overall, this led to training five pairs of models, such that each pair was composed of one LR trained on the NT and ESM2 model outputs and an isotonic calibrator trained on the LR’s predicted probabilities.

**Figure 2.**
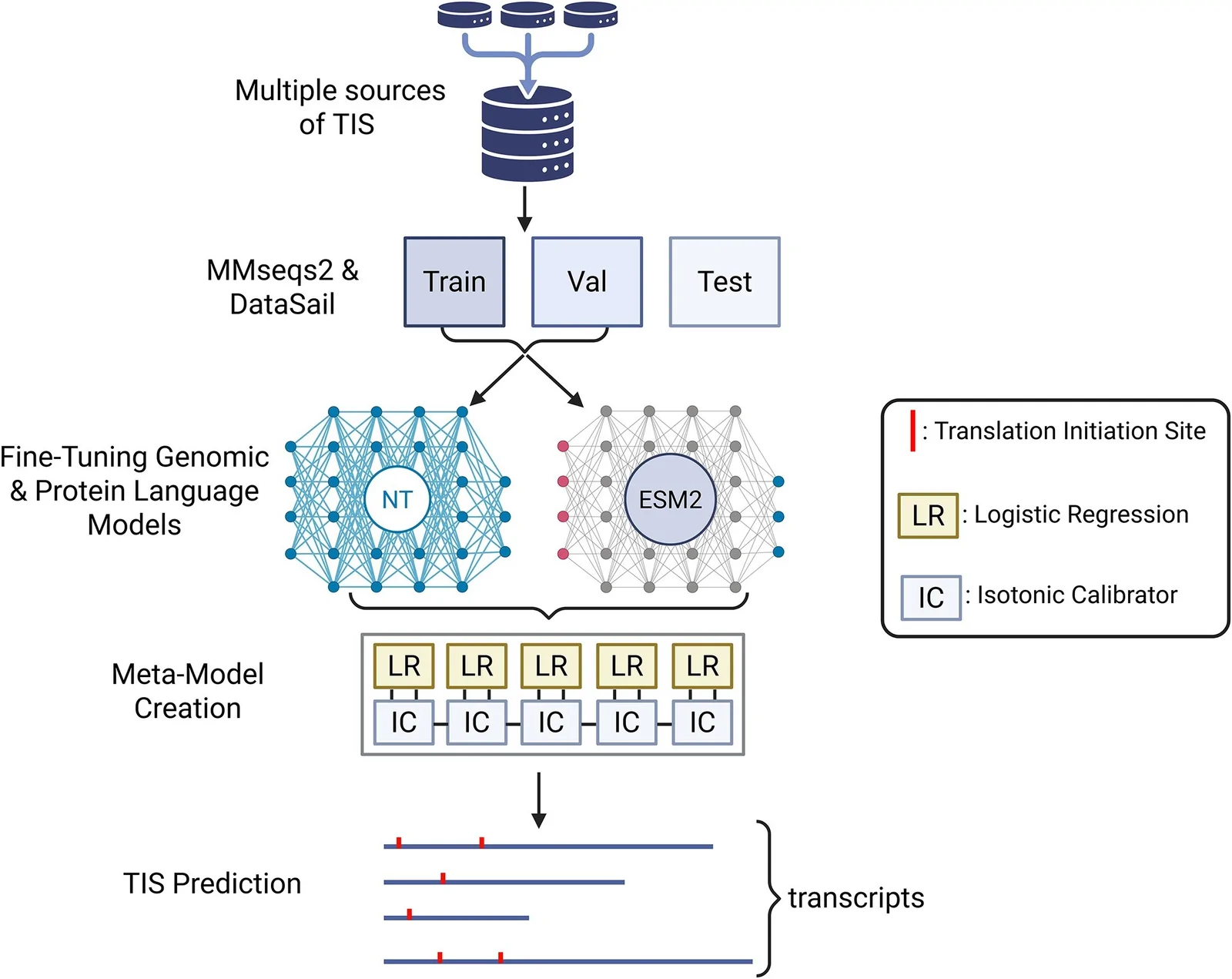
Workflow used to create MetaTIS by combining predictions from the NT and ESM2 models. The meta-model is composed of five pairs of LR models and their corresponding isotonic calibrators. The final TIS predictions are made by averaging the output of the five isotonic calibrators. This figure was created in BioRender. Schmitz, A. E. (2026) https://BioRender.com/opn9h4l.

At prediction time, a new input first passes through the NT and ESM2 models to obtain prediction probabilities for possible TIS. Then the predictions from both models are provided as features to the five LR models to extrapolate the probabilities, which are then adjusted by their respective isotonic model. The final prediction is the average output of the five isotonic calibrators. Figure 2 shows this workflow.

Instead of the regular Brier score (BS), the Brier skill score (BSS) was employed as the primary metric to quantify the accuracy and reliability of the meta-model’s probabilistic predictions [37]. The reason for this is that BS is dependent on the prevalence of the positive class and consequently can be difficult to interpret when assessing the performance of a classifier on datasets having different class proportions [38]. In BSS, the BS is normalized to render it more interpretable. The formulas of BS and BSS are illustrated below:


\begin{eqnarray*}
\mathrm{ BS} = \frac{1}{N}\mathop \sum \limits_{i = 1}^N {\left( {{f}_i - {o}_i} \right)}^2,
\end{eqnarray*}

\begin{eqnarray*}
\mathrm{ BSS} = 1 - \frac{{\mathrm{ B}{\mathrm{ S}}_{\mathrm{model}}}}{{\mathrm{ B}{\mathrm{ S}}_{\mathrm{baseline}}}},
\end{eqnarray*}

where *N* is the total number of samples, ${f}_i$ is the forecasted probability from the model, and ${o}_i$ is the actual outcome.$\ \mathrm{ B}{\mathrm{ S}}_{\mathrm{model}}$ represents the BS of the model and $\mathrm{ B}{\mathrm{ S}}_{\mathrm{baseline}}$ is the BS of a naïve classifier that always predicts the prevalence of the positive class. A BSS value of 1 indicates perfect calibration, 0 means it is no better than predicting the prevalence, and a negative value means it is worse than the naïve baseline.

### Previous models

Our models were contrasted against TITER [18] and the Gleanson *et al.* models [19] based on the performance of these models on our test set and each model’s respective test set. The performances of TranslationAI [23] and TRANSAID [24] were also reported on these three test sets. In each test set, the performances were computed solely based on the AUG, CUG, GUG, and UUG codons, as the Gleason *et al.* models are limited to these start codons.

The test sets defined by Gleason *et al.*, were retrieved from Github, featuring an AUG test set consisting of 172 positive and 172 negative instances; see https://github.com/Agleason1/TIS-Predictor. Sequences possessing missing nucleotide positions, represented as “N” in the Gleason *et al.* test set, were excluded of which 85 negative samples were incomplete. However, this test set only provides 10 upstream and 10 downstream flanking nucleotides, with respect to the TIS. Thus, the exact transcript sequences were retrieved by mapping the test sequences to the data sets used by Gleason *et al.* [8, 9]. As a result, 50 positives and 50 negatives could not be mapped with exact matches in this step. The final filtered AUG test set consisted of 122 positive and 72 negative instances. Subsequently, the near-cognate test set was used, consisting of 60 positive and 60 negative instances. As conducted beforehand, the first 32 negative instances with missing nucleotides were filtered out. Afterward, the same mapping was performed as described, yielding 37 positive and 20 negative near-cognate instances; the complete filtered Gleason *et al.* test set can be found in Supplementary Table S1.

The TITER test set was retrieved from Github, comprising of 767 positive and 9914 negative instances; see https://github.com/zhangsaithu/titer. The same processing steps used for the Gleason *et al.* test set were repeated, however mapping the sequences using solely the Gao *et al.* [8] data; as a result, 282 positive and 872 negative AUG instances, and 128 positive and 4609 negative near-cognate instances were obtained. In TITER [18], the negative TIS curation strategy is different, whereby only 10 non-TIS are selected that have the same triplet as the actual TIS within the transcript; the filtered TITER test set can be found in Supplementary Table S1.

Both the TITER and Gleason *et al.* test sets were created based on random splitting, which is prone to data leakage. Hence, the performances of TITER and Gleason *et al.* models on their own test sets could be inflated.

Additionally, our models’ and the NetStart2.0 [21] model’s performances were computed on the TISTransformer test set [20], composed of the annotated Ensembl transcripts present in chromosomes 1, 7, 13, and 19. The evaluation was based solely on the AUG predictions. Netstart2.0 was retrieved from https://github.com/lsandvad/netstart2.

As performance metrics, PRAUC and the area under the receiver operating characteristic curve (ROCAUC) were utilized; notably, both metrics are not threshold-specific and compensate for imbalance observed in the test sets.

### Data splitting

To avoid data leakage in our training and validation sets, MMseqs2 [27] was first employed to cluster the sequences, with a minimum sequence identity of 50%, minimum coverage of 80%, and –cov-mode 1. The TITER, Gleason *et al.*, and TISTransformer test set sequences were also included to remove any sequence that clusters with them from our data. After removing these sequences, DataSail’s identity-based splitting was used to create training, validation, and test sets based on the cluster annotations from MMseqs2 [28]. This led to having training, validation, and test sets composed of 43 822, 24 245, and 24 319 mRNA sequences, respectively. The number of TIS and non-TIS for the cognate and near-cognate (NC) cases in each set is detailed in Table 2. The distribution of positive start codons in each dataset is shown in Supplementary Fig. S1. The training, validation, and test datasets are available on https://huggingface.co/datasets/Arampapaz/MetaTIS_Train_Val_Test.

**Table 2.** Number of TIS and non-TIS in the training, validation, and test sets after splitting the sequences using MMseqs2 and DataSail

Dataset	AUG-TIS	NC-TIS	AUG-non-TIS	NC-non-TIS	non-TIS (all)
Train	34 390	17 826	1 535 475	13 060 323	89 100 354
Validation	19 209	9681	953 368	7 983 954	54 478 113
Test	19 692	9261	951 192	7 982 499	54 482 033

### Integrated gradients

To evaluate the contribution of the inputs to model outputs, the integrated gradients of each token, with respect to the actual TIS on the same transcript, were computed. To achieve this, the *IntegratedGradients.attribute* function from *Captum* version *0.8.0* was used, such that the baseline embedding was listed as an “*NNN*” token signifying any codon and the number of steps was set to 100 [39, 40].

### MRNA and protein expression correlation

Using the National Cancer Institute’s Clinical Proteomic Tumor Analysis Consortium (CPTAC), Python API transcriptomic and proteomic expression data were retrieved for matching patients with colon adenocarcinoma, uterine corpus endometrial carcinoma, and glioblastoma multiforme [41]. First, the Spearman correlation was calculated between the transcriptomic and proteomic expression data, followed by procuring the complementary DNA and 5′UTR sequences for each gene from Dyer *et al.* [11]. This was conducted with the aim of investigating whether genes that were predicted to have a translation-initiating AUG ORF by MetaTIS, prediction probability * \ge * 0.5, upstream of its canonical ORF, has lower correlation coefficients between transcriptomic and proteomic data when compared to genes predicted to have upstream AUG ORF that does not initiate translation, predicted probability * < * 0.5.

### MetaTIS webservice

A new webservice was developed to host our models and provide TIS predictions for any given transcript. It is freely available at https://service2.bioinformatik.uni-saarland.de/metatis/. The user should either provide the transcript ID, as the BioMart API [42] will fetch sequence information, or manually paste in the sequence to retrieve the predicted TIS. For manual submissions, the FASTA header should be omitted, and the nucleotide sequence should be provided as a continuous string without line breaks. This allows the analysis of non-standard transcripts that may not be available through BioMart.

All positions of the selected start codons will be provided with prediction probabilities. Therefore, the tool will provide predictions for multiple TIS across the reading frames. The results are visualized as a table containing information about the start codon, reading frame, start site, NT, ESM2, and MetaTIS predictions and can be downloaded as a csv file. Besides the MetaTIS predictions, we also provide the NT and ESM2 model predictions for the user to have an understanding about the outcomes based on the transcript-level or protein-level separately. The run time of the webserver was measured for different transcript lengths; see Supplementary Table S2. Additionally, a worked example of how to use the webserver was provided; see Supplementary Figs S2–S5.

## Results

### MetaTIS performance on AUG test sets

The competence of our models, the Gleason *et al.* models, TITER, TRANSAID, and TranslationAI was analyzed on multiple AUG test sets that exhibited varied negative curation strategies, class ratios, and TIS detection strategies. The results of this are illustrated in Table 3.

**Table 3.** Performances of the seven models on three different AUG test sets

Model	Internal test set (PRAUC baseline 0.02)	TITER test set (PRAUC baseline 0.24)	Gleason *et al.* test set (ROCAUC)
ESM2	0.85	0.90	0.95
NT	0.87	0.92	0.97
Meta	0.88	0.92	0.97
Gleason *et al.*	0.19	0.94	0.89
TITER	0.42	0.78	0.94
TRANSAID	0.83	0.90	0.92
TranslationAI	0.77	0.89	0.97

PRAUC was used on the internal and TITER test sets due to the true TIS being the minority class.

From Table 3, it is evident that all models were able to perform well across the test sets since all had performances significantly higher than the baseline. However, the biggest drop was seen in the Gleason *et al.* and TITER performances on the internal test set. This could be attributed to the fact that the models were trained on balanced subsampled data, which may lead to poor performance when the positive class, TIS, is very scarce. This was also seen in TITER’s performance in Clauwaert *et al.* [20]. The average precision scores of NT, ESM2, and MetaTIS for AUG TIS and non-TIS on the three test sets are found in Supplementary Table S3.

For further evaluation, the TISTransformer test set, composed of all annotated Ensembl transcripts present in chromosomes 1, 7, 13, and 19, was used, with only AUG candidate TIS sites considered. In table A2 of Clauwaert *et al.* [20], a PRAUC score of 0.83 was reported for the TISTransformer large model on this test set, while Netstart2.0 [21] was also applied on the same test set and achieved a PRAUC score of 0.77. Ultimately, our fine-tuned ESM2 model, NT model, and Meta-model reported superior PRAUC scores of 0.89, 0.91, and 0.92, respectively, for the TISTransformer test set. Overall, these findings validate our models’ ability to accurately identify AUG TIS across heterogeneous datasets without the confounding effects of data leakage.

### MetaTIS performance on CUG, GUG, and UUG test sets

For the near cognate cases, the Gleason *et al.* model was only trained on the CUG, GUG, and UUG instances. Therefore, we only kept these codons in our internal test set. This resulted in having 4734 TIS and 3 248 033 non-TIS. The model performances are shown in Table 4.

**Table 4.** Performances of the seven models on three different near-cognate test sets

Model	Internal test set (PRAUC baseline 0.001)	TITER test set (PRAUC baseline 0.027)	Gleason *et al.* test set (ROCAUC)
ESM2	0.41	0.07	0.71
NT	0.59	0.10	0.84
Meta	0.61	0.11	0.78
Gleason *et al.*	0.005	0.23	0.89
TITER With Prior	0.01	0.17	0.79
TITER Without Prior	0.01	0.19	0.76
TranslationAI	0.01	0.04	0.65

PRAUC was used on the internal and TITER test sets due to the true TIS being the minority class. Here, TRANSAID was not used since it only predicts AUG cases.

From Table 4, it is evident that all models demonstrated superior performance on AUG cases, as opposed to non-canonical cases. This suggests that sequence information is more crucial in AUG cases. Another possibility could be that our models are biased toward AUG TIS since the number of near-cognate TIS is much lower than AUG TIS. To test this, a near-cognate only NT model with the same parameters was trained and this led to a similar result on the internal non-AUG test set with a PRAUC of 0.597 compared to a PRAUC of 0.594 from the current general model. This suggests that the focal loss is able to properly handle the codon imbalance. The average precision scores of NT, ESM2, and MetaTIS for near-cognate TIS and non-TIS on the three test sets are found in Supplementary Table S4.

It is evident that NT boasted a higher performance than ESM2, whereby ESM2, by design, cannot differentiate between multiple codons coding for the same amino acid. TITER and Gleason *et al.* performed well on their test sets, but poorly on the internal test set, which is composed of TIS detected by different experimental approaches. This may reflect that in both studies the test sets were created using random splitting, which is prone to data leakage and often leads to inflated results. Additionally, a methodological bias can exist since both studies relied mostly on QTI-seq data [8] rather than incorporating alternative TIS detection approaches.

To test the effect of the TIS detection methodology on model predictions, we examined near-cognate TIS from the QTI-seq dataset [8] present in our internal test set. We identified 1075 near-cognate TIS instances and 144 704 non-TIS instances (random baseline PRAUC: 0.007). The NT model achieved a PRAUC of 0.18 on these cases, which is meaningfully above baseline, but notably lower than its overall performance on all near-cognate instances in the internal test set (PRAUC: 0.59). We attribute this gap primarily to the fact that QTI-seq detected TIS represent only a subset of the methodological diversity in our training data, meaning the model’s learned boundary is not optimally tuned for a single method. A consistent pattern was observed on the TITER test set, which also exclusively comprises TIS detected by QTI-seq (PRAUC: 0.10).

Tables 3 and 4 show that the most evident contributions of the ESM2 branch came from the non-AUG TIS predictions. We speculate that ESM2 can check whether the non-AUG translated protein is viable or not and may benefit the meta-model in that sense. To validate the benefit of incorporating the ESM2 outputs in the meta-model, we combined the predictions and labels from the internal, TITER, and Gleason *et al.* test sets of MetaTIS (Isotonic-NT-ESM2) for the non-AUG instances. Then we formed 200 bootstraps of this combined test set and computed the PRAUC of each bootstrap. The calculated PRAUC scores had a 95% confidence interval (CI) of [0.581, 0.611]. The same was done for the predictions of another meta-model that was trained solely on the NT model outputs (Isotonic-NT-only). On this combined test set, the PRAUC scores of this model had a 95% CI of [0.565, 0.592]. In summary, it was found that the Isotonic-NT-ESM2 had significantly higher PRAUC scores on the bootstrapped samples compared to the Isotonic-NT-only model (Mann–Whitney *U*-test *P*-value <.001).

### Calibration of model predictions

To further assess the models, we computed their Brier skill score on the three test sets. The BSS was also calculated for different calibration approaches to compare against the isotonic meta-model. These include Platt scaling [43] and regular LR fitted on the NT and ESM2 model outputs based on the validation set. They were fitted with either setting the class weight to balanced (-balanced) or without (-normal). We also checked the BSS of the NT, ESM2, TITER, and Gleason *et al.* models. The results are presented in Table 5.

**Table 5.** Brier skill score of the different models on the internal, TITER, and Gleason *et al.* test sets

Model	Internal test set	TITER test set	Gleason *et al.* test set
Isotonic-balanced	0.69	0.51	0.01
Isotonic-normal	0.64	0.51	0.02
Platt-balanced	0.68	0.51	−0.005
Platt-normal	0.64	0.51	0.02
LR-balanced	−15.13	−2.78	0.21
LR-normal	0.64	0.51	−0.004
NT	0.17	0.48	0.22
ESM2	0.24	0.47	0.08
TITER	−13.33	−1.22	0.46
Gleason *et al.*	−48.83	−2.49	0.35

“-balanced” represents the models that used a balanced class weights during fitting and the “-normal” represents the default fit.

Table 5 reports the BSS values for each model, where a BSS value of 1 indicates perfect calibration, 0 means it is no better than predicting the prevalence, and a negative value means it is worse than the naïve baseline. It is evident that the TITER and Gleason *et al.* models are worse than the naïve baseline on the internal and TITER test sets. The Isotonic-balanced model, which represents MetaTIS, had the highest BSS on the internal test set and equally highest on the TITER test set. MetaTIS is slightly better than the naïve baseline on the Gleason *et al.* test set. This is mainly due to the different class proportions that the isotonic model was trained on in the validation set. We further checked whether overfitting can be a possible explanation for this by training simpler models like Platt scaling and LR. However, these produced relatively similar results on the Gleason *et al.* test set suggesting that the main reason for the drop in performance is the different and non-representative class proportions seen in the Gleason *et al.* test set. Interestingly, the NT model had the highest BSS on the Gleason *et al.* test set among all our models. The calibration curves for MetaTIS on these three test sets are shown in Supplementary Fig. S6.

To support diverse use cases, the webserver reports predictions from all three models to the user. Users working in contexts where nucleotide-level or protein-level signals are more informative can refer to the individual outputs accordingly. This can be also done via the stand-alone code on Github.

### Importance of the Kozak sequence and upstream open reading frames

**Figure 3.**
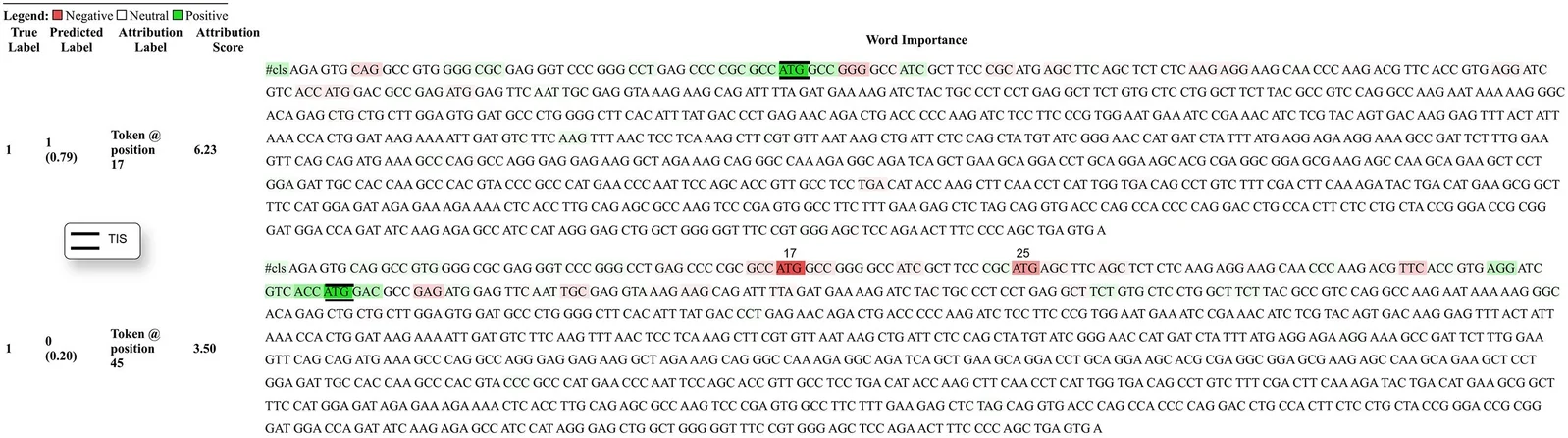
Token attribution scores based on the fine-tuned NT model outputs for tokens at positions 17 and 45, respectively. Tokens highlighted in green represent positive attributions, which influence the model to output a positive prediction, while tokens highlighted in red represent negative attributions and decrease the likelihood of a positive prediction. For the predicted label, a threshold of 0.5 was used.

Integrated gradients of the fine-tuned NT model were computed for each token, with respect to the actual TIS token on the same transcript. This allows for the evaluation of each codon’s contribution to the predicted output. The gradients were solely computed on positive instances in the internal test set. An example output is shown in Fig. 3 for the predictions on the NF2 gene, which exemplifies that the model strongly considers the start codon itself as relevant for its output whereby in both predictions, AUG encourages a positive prediction; this is expected, as most positive predictions feature an AUG start codon.

Furthermore, in both outputs, the model considered the first upstream and downstream tokens to contribute positive attributions toward an actual TIS. These tokens follow the Kozak pattern, such that there exists an “A/G” at the −3 position and a “G” at the +1 position. For the token at position 45, the model considered the two upstream AUGs to provide a strong negative attribution. This likely suggests that the model understands that translation is less likely to occur at position 45, as there exist other candidate start codons upstream. Notably, the AUG at position 17 featured a greater negative attribution on the model output when compared to the AUG at position 25, which could be attributed to the stronger sequence context present in the flanks of position 17 compared to the flanks of position 25.

To further check the influence of an upstream TIS on a TIS position downstream, we analyzed all transcripts in the internal test set that had multiple detected TIS. Given that all start codons possessed a median negative attribution score on a downstream TIS prediction, with the exception of AAG, which had a median attribution score close to zero, we can conclude that the model considers upstream TIS to negatively influence TIS positions downstream.

The integrated gradients of the NT model provide attribution scores for 3-mer tokens. To approximate and analyze whether the model captured the importance of the Kozak sequence at a single-nucleotide level, the attribution scores of tokens directly upstream of the TIS starting with A/G, which reflects the −3 position with respect to the TIS, were compared against all other tokens in the positive instances of the internal test set. The comparison was performed using the Mann–Whitney *U*-test with a significance threshold of 0.05. For the AUG TIS, the tokens starting with an A/G had a significantly higher median attribution score of 0.21 compared to the other tokens, which had a median of 0.046 (*P*-value <2.22 × 10^−16^). For the near-cognate TIS, we found that the tokens directly upstream of the TIS that start with an A/G had significantly higher median attribution scores than other tokens for only the CUG, GUG, AUU, AUC, and AUA TIS codons.

We also analyzed the tokens directly downstream and compared the attribution scores of the ones starting with a G, which reflects the +4 position, to the others. For the AUG TIS, the tokens starting with a G had a significantly higher median attribution score of 0.26 compared to the other tokens, which had a median of 0.09 (*P*-value <2.22 × 10^−16^). For the near-cognate TIS, we found that the tokens starting with a G had a significantly higher median attribution score only for the CUG, GUG, AAG, UUG, AUC, and ACG TIS codons. These results illustrate that the model correctly captures and differentiates the importance of the Kozak consensus sequence based on the start codon.

The importance of the start codon itself was also analyzed for all positive instances in the internal test set; see Supplementary Fig. S7. The fine-tuned NT model additionally assesses the prevalence of certain start codons over others; as expected, the model predicted AUG to harbor the highest positive attribution toward a true TIS and non-canonically, UUG displayed the highest median attribution score.

### Integrated gradients reveal the importance of an upstream region

In addition to the Kozak region, we also investigated the flanking region further upstream of the TIS. Here, the −4 to −15 nucleotide positions with respect to the TIS were considered. Interestingly for both the AUG and near-cognate cases, the presence of an adenine, A, at positions −12, −11, or −10 contributed very positively toward translation initiation. For AUG, adenine had median attributions of 0.32, 0.15, and 0.21 for positions −12, −11, and −10, respectively. For the near-cognate cases, it had median attributions of 0.54, 0.38, and 0.25 for positions −12, −11, and −10, respectively. The reason for these high attribution scores could be that an adenine-rich stretch in this window is less likely to form stable secondary structures, keeping the mRNA single-stranded, and accessible for smooth ribosomal scanning that allows easier engagement with the TIS.

We further analyzed the cumulative magnitude of the attribution scores of the upstream tokens with respect to the TIS predictions. This was performed by taking the median of the absolute values of the attribution scores for each upstream token. For both the cognate and near-cognate TIS, we found that the token that is four positions upstream of the TIS token, which represents the −12, −11, and −10 nucleotide positions, to have the highest median absolute attribution with median absolute attributions of 0.22 and 0.27 for the AUG and near-cognate cases, respectively. The second best in both cases was the first upstream token, which represents the −3, −2, and −1 nucleotide positions. This indicates that the model predictions for TIS are more strongly influenced by positions upstream of the well-studied Kozak region. The results of the integrated gradients are provided in Supplementary Table S5.

### MRNA and protein expression correlation

Using the Spearman correlations between mRNA and protein levels for colon adenocarcinoma, uterine corpus endometrial carcinoma, and glioblastoma multiforme samples, as well as the corresponding sequence information, possible AUG TIS were predicted in the 5′UTRs of several genes. The purpose of this analysis is that an active TIS upstream of a gene’s annotated TIS may disrupt the main ORF initiation, leading to lower correlations between mRNA and protein levels of that gene. For colon adenocarcinoma, 2269 genes were analyzed; 109 of which were found to have a translation-initiating AUG upstream of their canonical start site by MetaTIS, with a median Spearman correlation of 0.27, while the remaining genes possessed a correlation of 0.31. For uterine corpus endometrial carcinoma, 3676 genes were investigated, with 168 of them found to possess an upstream AUG ORF that initiates translation, with a median correlation coefficient of 0.4; the remainder of the genes possessed a correlation of 0.43. Lastly, for the glioblastoma multiforme data, 4132 genes were investigated, with MetaTIS predicting 184 genes to possess an upstream AUG TIS, with a median correlation coefficient of 0.43; the remainder of genes output a median correlation of 0.46.

We then combined all the model predictions for the three cancer types and performed a correlation analysis between the MetaTIS prediction probabilities for an AUG uORF and the mRNA and protein expression correlations. A Spearman correlation of −0.103 with a *P*-value <.001 was found. The correlation coefficient is significantly negative, indicating that the higher the model prediction for an uORF the lower is the correlation between that transcript’s expression and its protein.

### Detection of uORFs

A weak correlation between a transcript and its protein is not always explained by the presence of an uORF. Thus, we performed an additional analysis to further test the ability of our model in detecting both AUG and non-AUG uORFs, for all nine near-cognate codons. To this end, we gathered all human uORF information from uORFdb [44], a comprehensive database for eukaryotic uORFs, and retrieved their transcripts through Biomart API [42]. This led to us having 18 918 transcripts with 23 005 AUG and 448 964 near-cognate uORFs. We then identified non-TIS instances by considering AUG and near-cognate positions in the transcripts that were not annotated by uORFdb and made sure to exclude the canonical TIS. This led to having 1 037 858 AUG and 8 544 313 near-cognate non-TIS. We then fed the transcripts to MetaTIS to get prediction probabilities. For the AUG cases, a ROCAUC score of 0.96 and a PRAUC of 0.39 were obtained. For the near-cognate cases, a ROCAUC score of 0.89 and a PRAUC of 0.36 were obtained. Overall, these results highlight the ability of our model to predict both cognate and near-cognate uORFs.

## Discussion

In contemporary transcriptomics, it is crucial to detect putative non-canonical TIS since these have been shown to regulate gene expression in both healthy and disease conditions, such as colorectal cancer [45, 46]. In particular, cancer cells have been shown to use non-canonical translation initiation to aid in their survival and developmental progression into malignancy [47]. A recent study has illustrated how targeting uORFs with mitotic inhibitors may enhance immune recognition and tumour cell elimination [48]. However, verifying such initiation sites requires the use of costly sequencing techniques, such as ribosome profiling paired with mass spectrometry-based methods, to confirm the presence of translated proteins. To surmount this, all annotated human TIS from Ensembl GRCh38.p14 [11] were curated here and combined with experimentally verified alternative TIS sites from five previous studies [8–10, 16, 29] in order to fine-tune two biological foundation models for TIS prediction. Ultimately, our models demonstrated formidable performance across four distinct test sets for both AUG and near-cognate TIS, as shown in Tables 3 and 4. Unlike in previous studies [18, 19], the test sets were intentionally generated with measures to mitigate data leakage, using a combination of MMseqs2 [27] and DataSail [28]. Observable variation in absolute performance was still present across test sets, which is attributed to varying class proportions, negative curation strategies, and experimental TIS detection methodologies.

To further interpret fine-tuned NT model outputs, the integrated gradients approach was implemented to compute the contribution of each codon on the TIS token output. The NT model was employed due to its superior performance compared to the ESM2 model, with the 3-mer nucleotide tokenization allowing for a refined view of the sequence context. When performed on positive instances of the internal test set, it was found that in order for the model to consider a strong Kozak sequence context [2] as a contributor toward a positive prediction, there must exist an A/G at the −3 position and a G at the +1 position, with respect to the TIS. Additionally, due to the token classification fine-tuning approach, the model can correctly consider an upstream TIS to attenuate downstream TIS, as reported in Andreev *et al.* [32]. Besides the Kozak pattern, the model also considered the −12 to −10 region to be crucial in determining whether a position is a TIS or not. In fact, it considered the presence of adenines at this region to be beneficial for translation to occur. An explanation for this could be that an adenine-rich stretch in this window disfavours formation of stable secondary structures, keeping the mRNA single-stranded, which allows the ribosome to easily engage with the TIS.

The novel meta-model, MetaTIS, was developed to provide calibrated predictions inferring the true probability of finding a TIS at a certain position in sequence. This was achieved by training five pairs of models, such that each pair was composed of one LR trained on the NT and ESM2 model outputs and an isotonic calibrator trained on the LR’s predicted probabilities. MetaTIS not only demonstrated that it ranks positive predictions more highly than negative ones across test sets, but is also able to maintain strong calibrations, with the only exception being the Gleason *et al.* test set, where the Brier skill score was slightly better than the baseline; see Tables 3, 4, and 5. The saliency of the MetaTIS predictions were further investigated on CPTAC data. Whereby, its predictions were utilized to detect upstream AUG ORFs on several genes in colon adenocarcinoma, uterine corpus endometrial carcinoma, and glioblastoma multiforme that alter and regulate the translation of the main ORF [49–52]. MetaTIS was also able to predict correctly both cognate and near-cognate uORFs annotated in uORFdb [44].

A limitation of our approach is that sequence information alone is not the only factor regulating translation initiation. In fact, multiple eIFs are required for the translation to initiate [53, 54]. Additionally, well-known uORFs are mostly initiated under certain conditions, such as stress, amino acid scarcity, and high cell density, among others. These conditions are associated with a phenomenon known as “leaky scanning,” in which the ribosome skips the initiation codon and continues scanning downstream in the sequence [55]. Without the consideration of these eIFs and cellular conditions, machine learning classifiers cannot make condition-specific predictions.

In summary, three models were developed to predict both canonical and non-canonical translation initiation start sites in human for all three reading frames in a transcript. Predictions for a certain transcript can be obtained using the following webserver: https://service2.bioinformatik.uni-saarland.de/metatis/. For larger computations, the models can be used with the scripts available on https://github.com/AramPapaz/MetaTIS. We believe that these models will help in identifying crucial cognate and near-cognate TIS that act as important regulators for certain diseases or in different developmental contexts.

## Supplementary Material

lqag100_Supplemental_Files

## Data Availability

Data are available on https://doi.org/10.5281/zenodo.19609644.
