# Supplementary material for: MetaTIS: a tool to predict cognate and near-cognate translation initiation sites in human
Source: NAR Genom Bioinform. 2026 Aug 24;8(3):lqag100. doi: 10.1093/nargab/lqag100 (PMC13501135; doi:10.1093/nargab/lqag100)
Supplement: Supplementary file 1 [file lqag100_supplemental_files.zip › Supplementary.docx]

Supplementary

**Figure S1:** True TIS codon distributions in the train, validation, and test sets.

**MetaTIS Webserver**

To use the webserver the user must first go to the Predict tab as highlighted in Figure S2.

**Figure S2:** The MetaTIS webserver homepage with the *Predict* tab highlighted in red.

Then the user should either provide the transcript ID, as the BioMart API will fetch sequence information in the red highlighted region of Figure S3, or manually paste the sequence to retrieve the predicted translation initiation sites in the green highlighted region of Figure S3. For manual submissions, the FASTA header should be omitted, and the nucleotide sequence should be provided as a continuous string without line breaks. This allows the analysis of non-standard transcripts that may not be available through BioMart.

**Figure S3:** The MetaTIS webserver’s *Predict* tab with the transcript ID and sequence sections highlighted in red and green respectively.

Once the sequence is provided the user must select which out of the ten possible start codons they would like predictions for. This is highlighted in Figure S4.

**Figure S4:** The MetaTIS webserver’s *Predict* tab with the sequence section highlighted in red. The sequence is provided and eight out of the ten start codons have been selected for predictions.

Now the user should press submit and the transcript will be passed as input to the models. The time it will take to get the predictions will vary depending on the transcript size and the number of start codons selected. To analyze this, we computed the run time of the webserver on transcripts having 100 to 10000 nucleotides, see table S2. The ten start codons were selected for each run, which represents the worst case. The runtimes are listed as the mean run time in seconds ± the standard deviation after five runs.

| **Transcript Length** | **Runtime (seconds)** |
| --- | --- |
| 100 | 0.34±0.04 |
| 1000 | 0.94±0.03 |
| 10000 | 19.76±0.22 |

**Table S2:** Mean runtime in seconds ± standard deviation after five runs for each transcript on the MetaTIS webserver.

In the results table, all positions of the selected start codons will be provided with prediction probabilities. The tool will provide predictions for multiple TIS across the reading frames. The table contains information about the start codon, reading frame, start site, NT, ESM2, and MetaTIS predictions and can be downloaded as a csv file. Note that besides the MetaTIS predictions, we also provide the NT and ESM2 model predictions to the user to have an understanding about the outcomes based on the transcript-level or protein-level separately. This table can be downloaded as a csv file. An example of the output table is shown in Figure S5.

**Figure S5:** The MetaTIS webserver’s output table with the rows sorted based on the MetaTIS prediction probability.

**Model Average Precision Scores**

| Test Set | NT | ESM2 | Meta |
| --- | --- | --- | --- |
| Internal-TIS | 0.88 | 0.85 | 0.88 |
| Internal-non-TIS | 0.99 | 0.99 | 0.99 |
| TITER-TIS | 0.92 | 0.90 | 0.92 |
| TITER-non-TIS | 0.98 | 0.97 | 0.98 |
| Gleason-TIS | 0.99 | 0.98 | 0.98 |
| Gleason-non-TIS | 0.95 | 0.87 | 0.94 |

**Table S3:** Average precision scores across test sets for AUG cases.

| Test Set | NT | ESM2 | Meta |
| --- | --- | --- | --- |
| Internal-TIS | 0.59 | 0.41 | 0.61 |
| Internal-non-TIS | 0.99 | 0.99 | 0.99 |
| TITER-TIS | 0.11 | 0.07 | 0.11 |
| TITER-non-TIS | 0.99 | 0.98 | 0.99 |
| Gleason-TIS | 0.85 | 0.80 | 0.85 |
| Gleason-non-TIS | 0.7 | 0.58 | 0.70 |

**Table S4:** Average precision scores across test sets for near-cognate cases.

**Figure S6:** Calibration curves based on the meta model predicted probabilities on the (a) internal, (b) TITER, and (c) Gleason et al. test sets. The dashed diagonal line plots a perfect calibration curve.

**Figure S7:** Distribution of attribution scores of the start codon on the output of the fine-tuned NT model, for all positives instances in the internal test set.
